# Supplementary material for: Prevalence of visual impairment due to refractive error among children and adolescents in Ethiopia: A systematic review and meta-analysis
Source: PLoS One. 2022 Aug 18;17(8):e0271313. doi: 10.1371/journal.pone.0271313 (PMC9387832; doi:10.1371/journal.pone.0271313)

**Supporting information 2- search terms used in different data base for visual impairment due to refractive error in Ethiopia.**

**Searching terms used**

1. **PubMed**-search strategy

Search: ((((("refractive error") OR (amblyopia)) OR (astigmatism)) OR (hyperopia)) AND (children)) AND (Ethiopia)


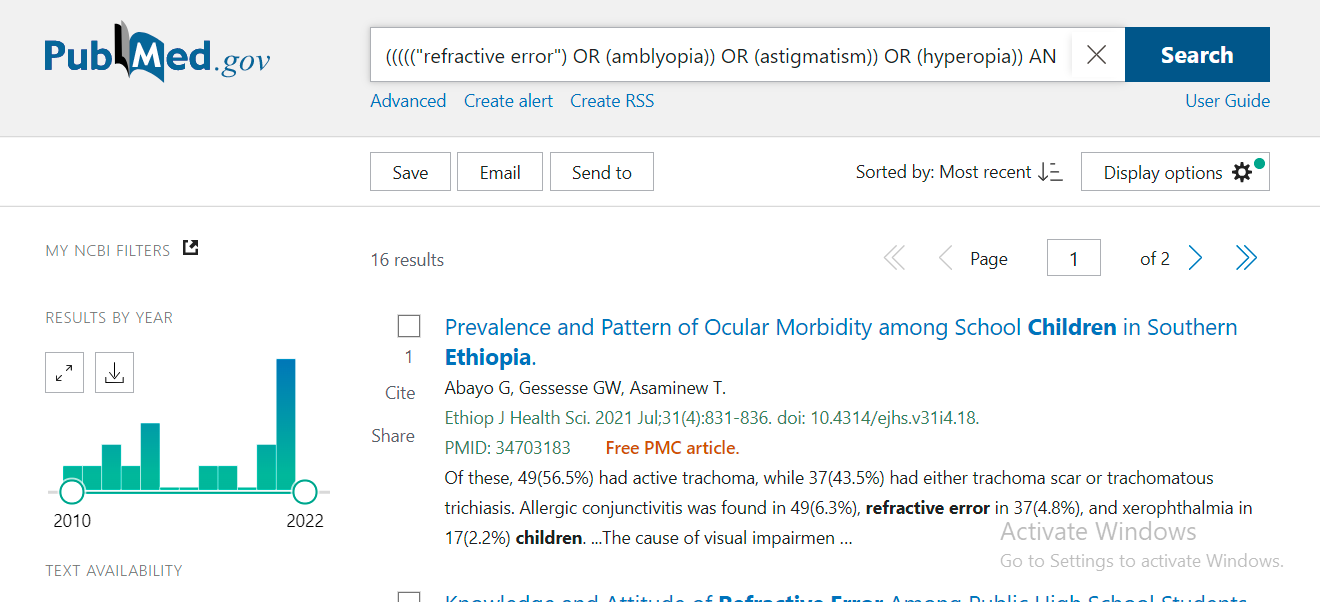


1. Science direct

“Refractive error” AND “Visual impairment” AND Ethiopia


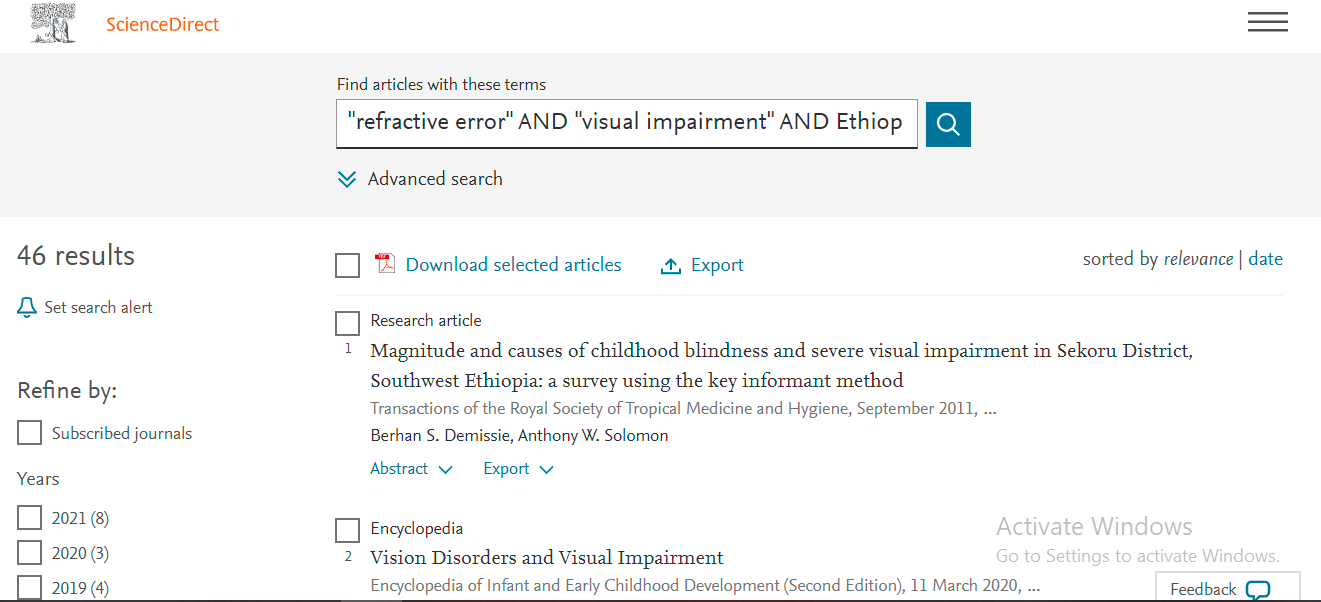


1. **HINARI**

("Refractive error") AND (“Visual impairment”) AND (Ethiopia) – 225 (17)


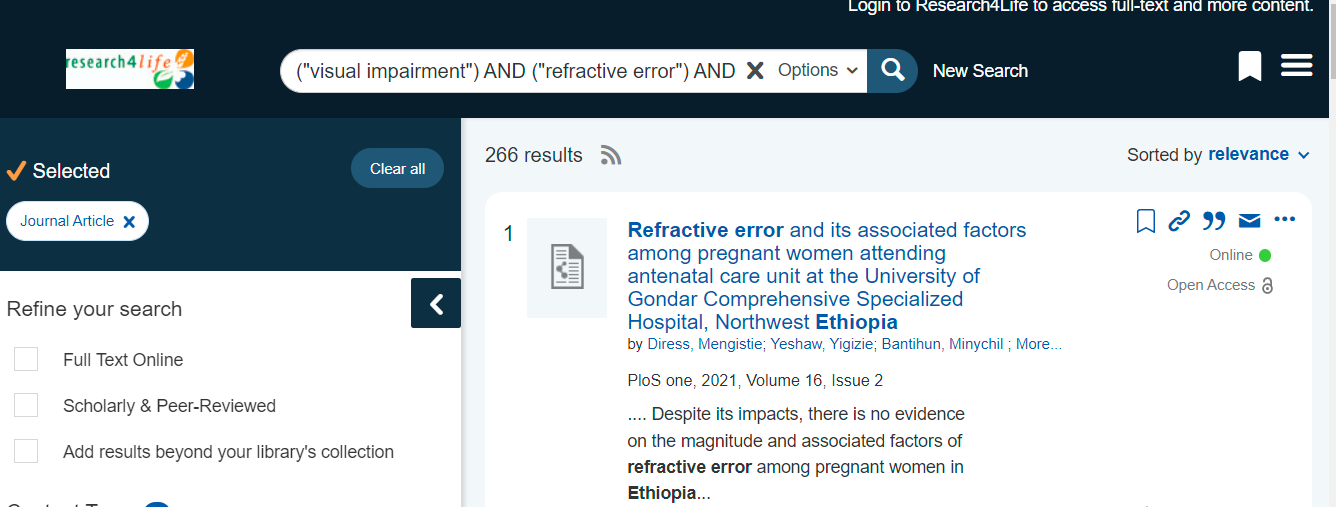


1. POPLINE

("Refractive error") AND (“Visual impairment”) AND (Ethiopia)


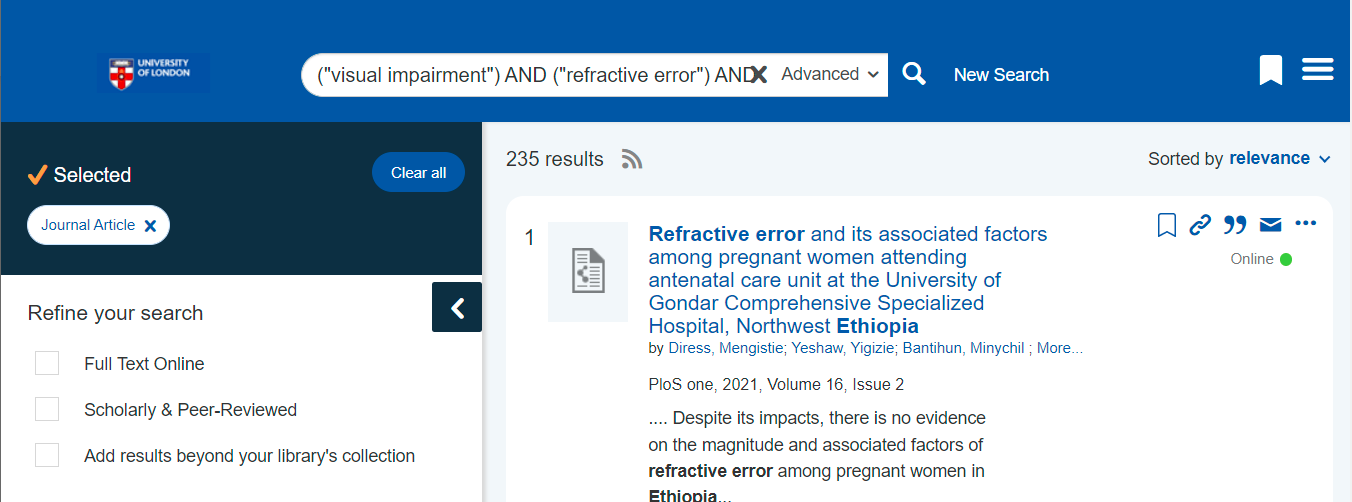


1. **Scopus**

Visual AND Impairment AND Refractive AND error AND Ethiopia


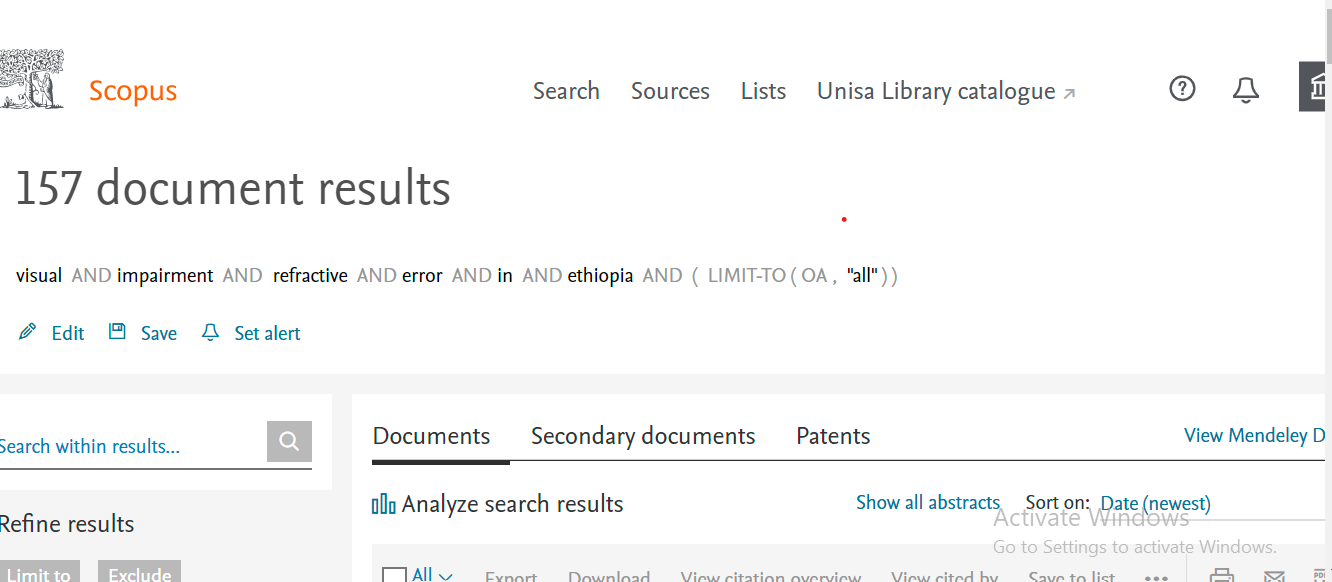


1. Google scholar

**"Refractive error" AND "visual impairment" AND Ethiopia**


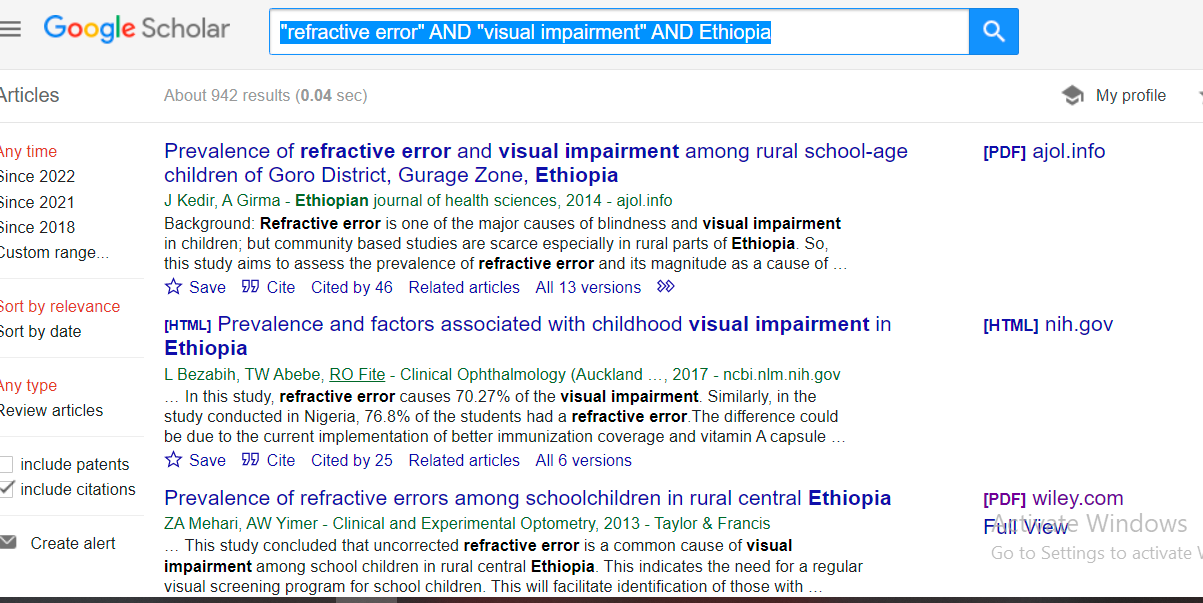

Supplement: S1 File — (DOCX) [file pone.0271313.s002.docx]
