# Supplementary material for: Prevalence of visual impairment due to refractive error among children and adolescents in Ethiopia: A systematic review and meta-analysis
Source: PLoS One. 2022 Aug 18;17(8):e0271313. doi: 10.1371/journal.pone.0271313 (PMC9387832; doi:10.1371/journal.pone.0271313)
Supplement: S1 Table — (DOCX) [file pone.0271313.s003.docx]

Supporting information 3- Table: showing risk of bias assessment of included study for metanalysis of visual impairment in Ethiopia.

| Author name | Year of publication | Q1 | Q2 | Q3 | Q4 | Q5 | Q6 | Q7 | Q8 | Q9 | Total score | Risk of Bias |
| --- | --- | --- | --- | --- | --- | --- | --- | --- | --- | --- | --- | --- |
| Zelalem et al (29). | 2019 | 0 | 0 | 0 | 0 | 0 | 0 | 1 | 0 | 0 | 1 | low |
| Sewunet et al(21). | 2014 | 0 | 0 | 0 | 0 | 0 | 0 | 0 | 0 | 0 | 0 | low |
| Kassa and Alene (30) | 2004 | 0 | 0 | 0 | 0 | 0 | 1 | 0 | 0 | 0 | 1 | low |
| Weldeamanuel et al(8). | 2020 | 0 | 0 | 0 | 0 | 0 | 0 | 0 | 0 | 0 | 0 | low |
| Alem and Gebru(9) | 2021 | 0 | 0 | 0 | 0 | 0 | 0 | 0 | 0 | 0 | 0 | low |
| Darge et al(31). | 2017 | 0 | 1 | 0 | 0 | 0 | 1 | 0 | 1 | 0 | 3 | medium |
| Ferede et al(32). | 2020 | 0 | 0 | 0 | 0 | 0 | 1 | 1 | 0 | 1 | 3 | medium |
| Assem et al(33). | 2021 | 0 | 0 | 0 | 0 | 1 | 1 | 0 | 1 | 1 | 4 | medium |
| Yared et al(34). | 2012 | 0 | 0 | 0 | 0 | 0 | 0 | 0 | 0 | 1 | 1 | low |
| Bezabih et al (35). | 2017 | 0 | 1 | 0 | 1 | 0 | 0 | 0 | 1 | 1 | 4 | medium |
| Hailu et al(17). | 2020 | 1 | 0 | 0 | 1 | 0 | 0 | 0 | 1 | 1 | 4 | medium |
| Mehari and Yimer(18). | 2012 | 0 | 0 | 0 | 1 | 0 | 1 | 1 | 0 | 1 | 4 | medium |
| Kedir and Girma(20). | 2010 | 0 | 0 | 0 | 0 | 0 | 1 | 0 | 0 | 0 | 1 | low |
| Dhanesha et al(21). | 2018 | 0 | 0 | 0 | 1 | 0 | 0 | 0 | 0 | 1 | 2 | low |
| Mehari et al (28). | 2014 | 0 | 0 | 0 | 0 | 1 | 0 | 0 | 1 | 0 | 2 | low |
| Shaffi and Bejiga(27). | 2005 | 0 | 0 | 0 | 0 | 0 | 1 | 0 | 0 | 1 | 2 | low |
| Demissie and Demissie(24). | 2014 | 0 | 0 | 0 | 0 | 0 | 0 | 0 | 0 | 1 | 1 | low |
| Belete et al(25). | 2016 | 0 | 0 | 0 | 0 | 0 | 0 | 0 | 1 | 0 | 1 | low |
| Gessese and Teshome(26). | 2020 | 0 | 0 | 0 | 0 | 0 | 0 | 0 | 0 | 0 | 0 | low |
| Demissie et al(23). | 2011 | 0 | 0 | 0 | 0 | 0 | 1 | 0 | 0 | 1 | 2 | low |
| Asferaw et al(22). | 2017 | 0 | 0 | 0 | 0 | 0 | 0 | 0 | 1 | 0 | 1 | low |
| Tegegne et al(19). | 2021 | 1 | 0 | 0 | 1 | 0 | 0 | 0 | 0 | 0 | 2 | low |
| **Q1 = Was the sample frame appropriate to address the target population?** | | | | | | | | | |  |  |  |
| **Q2 = Were study participants sampled in an appropriate way?** | | | | | | | | | |  |  |  |
| **Q3 = Was the sample size adequate?** | | | | | | | | | |  |  |  |
| **Q4 = Were the study subjects and the setting described in detail?** | | | | | | | | | |  |  |  |
| **Q5 = Was the data analysis conducted with sufficient coverage of the identified sample?** | | | | | | | | | |  |  |  |
| **Q6 = Were valid methods used for the identification of the condition?** | | | | | | | | | |  |  |  |
| **Q7 = Was the condition measured in a standard, reliable way for all participants?** | | | | | | | | | |  |  |  |
| **Q8 = Was there appropriate statistical analysis?** | | | | | | | | | |  |  |  |
| **Q9 = Was the response rate adequate, and if not, was the low response rate managed appropriately?** | | | | | | | | | |  |  |  |
