# Supplementary material for: Prevalence of visual impairment due to refractive error among children and adolescents in Ethiopia: A systematic review and meta-analysis
Source: PLoS One. 2022 Aug 18;17(8):e0271313. doi: 10.1371/journal.pone.0271313 (PMC9387832; doi:10.1371/journal.pone.0271313)
Supplement: Attachment — Submitted filename: PLOS_PeerReviewWithComments.pdf [file pone.0271313.s004.pdf]

PLOS ONE

Prevalence of Visual Impairment due to Refractive Error among children and adolescents in Ethiopia: A Systematic Review and Meta-Analysis.

--Manuscript Draft--

Manuscript Number:	PONE-D-22-10704
Article Type:	Research Article
Full Title:	Prevalence of Visual Impairment due to Refractive Error among children and adolescents in Ethiopia: A Systematic Review and Meta-Analysis.
Short Title:	Prevalence of Visual Impairment due to Refractive Error among children and adolescents in Ethiopia
Corresponding Author:	Daniel Atlaw<br>Madda Walabu university<br>Goba, Oromia ETHIOPIA
Keywords:	visual, impairment, refractive, error, meta-analysis
Abstract:	Abstract<br>Introduction: Globally, the prevalence of refractive error was 12% (4), and visual impairment due to refractive error was 2.1%. In sub-Saharan Africa, the prevalence of refractive error and visual impairment due to refractive error was 12.6% and 3.4%, respectively. In Ethiopia, the prevalence of visual impairment due to refractive error varies from 2.5% in the Gurage zone to 12.3% in Hawassa city. Hence, this Meta-analysis aimed to summarize the pooled prevalence of visual impairment due to refractive error in Ethiopia.<br>Methods: A systematic search of the literature was conducted by the authors to identify all relevant primary studies. All articles on the prevalence of visual impairment due to refractive error in Ethiopia were identified through a literature search. The databases used to search for studies were PubMed, Science Direct, POPLINE, HENARI, Google Scholar, and grey literature was searched on Google until December 15, 2021. In this meta-analysis, the presence of publication bias was evaluated using funnel plots and Begg's tests at a significance level of less than 0.05. The sensitivity analysis was conducted to check for a single study's effect on the overall prevalence of refractive error.<br>Result: About 1664 studies were retrieved from initial electronic searches using international databases and google searches. A total number of 20,088 children and adolescents were included in this meta-analysis. The pooled prevalence of refractive error in Ethiopia using the random effect model was estimated to be 6 % (95% CI, 5-7) with a significant level of heterogeneity (I2 = 94.4 %; p < 0.001). The pooled prevalence of visual impairment due to refractive was analyzed by subtypes, and pooled prevalence was estimated to be 4%, 5.2%, and 1% for myopia, hyperopia, and astigmatism, respectively.<br>Conclusion : The pooled prevalence of visual impairment due to refractive error was high in Ethiopia. About one in twenty-five Ethiopian children and adolescents are affected by visual impairment due to refractive error.
Order of Authors:	Daniel Atlaw
	Zerihun Shiferaw
	Biniyam Sahiledengele
	Sisay Degno
	Ayele Mamo
	Demisu Zenbaba
	Habtamu Gezahegn
	Fikreab Desta
	Wogene Negash
	Tesfaye Assefa
	Mujib Abdela

Powered by Editorial Manager® and ProduXion Manager® from Aries Systems Corporation

	Abbul Haseno
	Gashaw Walle
	Chala Kene
	Degefa Gamora
	Vijay Kumar Chattu

Additional Information:	
**Question**	**Response**
**Financial Disclosure**<br><br>Enter a financial disclosure statement that describes the sources of funding for the work included in this submission. Review the submission guidelines for detailed requirements. View published research articles from *PLOS ONE* for specific examples.<br><br>This statement is required for submission and **will appear in the published article** if the submission is accepted. Please make sure it is accurate.<br><br>**Unfunded studies**<br>Enter: *The author(s) received no specific funding for this work.*<br><br>**Funded studies**<br>Enter a statement with the following details:<br>• Initials of the authors who received each award<br>• Grant numbers awarded to each author<br>• The full name of each funder<br>• URL of each funder website<br>• Did the sponsors or funders play any role in the study design, data collection and analysis, decision to publish, or preparation of the manuscript?<br>• **NO** - Include this sentence at the end of your statement: *The funders had no role in study design, data collection and analysis, decision to publish, or preparation of the manuscript.*<br>• **YES** - Specify the role(s) played.<br><br>* typeset	The authors received no specific funding for this work
**Competing Interests**<br><br>Use the instructions below to enter a competing interest statement for this submission. On behalf of all authors,	The authors have declared that no competing interests exist.

disclose any competing interests that could be perceived to bias this work—acknowledging all financial support and any other relevant financial or non-financial competing interests.<br><br>This statement is required for submission and **will appear in the published article** if the submission is accepted. Please make sure it is accurate and that any funding sources listed in your Funding Information later in the submission form are also declared in your Financial Disclosure statement.<br><br>View published research articles from *PLOS ONE* for specific examples.<br><br>**NO authors have competing interests**<br><br>Enter: *The authors have declared that no competing interests exist.*<br><br>**Authors with competing interests**<br><br>Enter competing interest details beginning with this statement:<br><br>*I have read the journal's policy and the authors of this manuscript have the following competing interests: [insert competing interests here]*<br><br>* typeset	
**Ethics Statement**<br><br>Enter an ethics statement for this submission. This statement is required if the study involved:<br><br>• Human participants<br>• Human specimens or tissue<br>• Vertebrate animals or cephalopods<br>• Vertebrate embryos or tissues<br>• Field research<br><br>Write "N/A" if the submission does not require an ethics statement.	N/A

Powered by Editorial Manager® and ProduXion Manager® from Aries Systems Corporation

General guidance is provided below. Consult the submission guidelines for detailed instructions. **Make sure that all information entered here is included in the Methods section of the manuscript.**<br><br>**Format for specific study types**<br><br>**Human Subject Research (involving human participants and/or tissue)**<br>• Give the name of the institutional review board or ethics committee that approved the study<br>• Include the approval number and/or a statement indicating approval of this research<br>• Indicate the form of consent obtained (written/oral) or the reason that consent was not obtained (e.g. the data were analyzed anonymously)<br><br>**Animal Research (involving vertebrate animals, embryos or tissues)**<br>• Provide the name of the Institutional Animal Care and Use Committee (IACUC) or other relevant ethics board that reviewed the study protocol, and indicate whether they approved this research or granted a formal waiver of ethical approval<br>• Include an approval number if one was obtained<br>• If the study involved *non-human primates*, add *additional details* about animal welfare and steps taken to ameliorate suffering<br>• If anesthesia, euthanasia, or any kind of animal sacrifice is part of the study, include briefly which substances and/or methods were applied<br><br>**Field Research**<br><br>Include the following details if this study involves the collection of plant, animal, or other materials from a natural setting:<br>• Field permit number<br>• Name of the institution or relevant body that granted permission	
**Data Availability**<br><br>Authors are required to make all data	Yes - all data are fully available without restriction

Powered by Editorial Manager® and ProduXion Manager® from Aries Systems Corporation

underlying the findings described fully available, without restriction, and from the time of publication. PLOS allows rare exceptions to address legal and ethical concerns. See the PLOS Data Policy and FAQ for detailed information.<br><br>A Data Availability Statement describing where the data can be found is required at submission. Your answers to this question constitute the Data Availability Statement and **will be published in the article**, if accepted.<br><br>**Important:** Stating 'data available on request from the author' is not sufficient. If your data are only available upon request, select 'No' for the first question and explain your exceptional situation in the text box.<br><br>Do the authors confirm that all data underlying the findings described in their manuscript are fully available without restriction?	
**Describe where the data may be found in full sentences. If you are copying our sample text, replace any instances of XXX with the appropriate details.**<br><br>• If the data are **held or will be held in a public repository**, include URLs, accession numbers or DOIs. If this information will only be available after acceptance, indicate this by ticking the box below. For example: *All XXX files are available from the XXX database (accession number(s) XXX, XXX.*).<br>• If the data are all contained **within the manuscript and/or Supporting Information files**, enter the following: *All relevant data are within the manuscript and its Supporting Information files.*<br>• If neither of these applies but you are able to provide **details of access elsewhere**, with or without limitations, please do so. For example:<br><br>*Data cannot be shared publicly because of [XXX]. Data are available from the*	All relevant data are within the manuscript and its Supporting Information files.

XXX Institutional Data Access / Ethics Committee (contact via XXX) for researchers who meet the criteria for access to confidential data.<br><br>*The data underlying the results presented in the study are available from (include the name of the third party and contact information or URL).*<br>• This text is appropriate if the data are owned by a third party and authors do not have permission to share the data.<br><br>* typeset	
Additional data availability information:	

Powered by Editorial Manager® and ProduXion Manager® from Aries Systems Corporation

Click here to access/download;Manuscript;manuscript on VI_VKC.docx

Prevalence of Visual Impairment due to Refractive Error among children and adolescents in Ethiopia: A Systematic Review and Meta-Analysis.

Daniel Atlaw[1]*, Zerihun Shiferaw[1], Biniyam Sahiledengele[2], Sisay Degno[3], Ayele Mamo[4], Demisu Zenbaba[2], Habtamu Gezahegn[1], Fikreab Desta[2], Wogene Negash[5], Tesfaye Assefa[5], Mujib Abdela[7], Abbul Haseno[1], Gashaw Walle[6], Chala Kene[7], Degefa Gamora[7] Vijay Kumar Chattu[8,9]

[1]Department of Biomedical Sciences, Madda Walabu University Goba Referral Hospital, Bale-Goba, Ethiopia

[2]Department of Public Health, Madda Walabu University Goba Referral Hospital, Bale-Goba, Ethiopia

[3]Department of Public Health, Madda Walabu University Shashemene Campus, Shashemene, Ethiopia

[4]Department of Pharmacy, Madda Walabu University Goba Referral Hospital, Bale-Goba, Ethiopia

[5]Department of Nursing, Madda Walabu University Goba Referral Hospital, Bale-Goba, Ethiopia

[6]Department of Biomedical Sciences, Debre Tabor University, Debre Tabor, Ethiopia

[7]Department of Midwifery, Madda Walabu University Goba Referral Hospital, Bale-Goba, Ethiopia

[8]Center for Transdisciplinary Research, Saveetha Institute of Medical and Technological Sciences, Saveetha University, Chennai 600077, India

[9]Department of Community Medicine, Faculty of Medicine, Datta Meghe Institute of Medical Sciences, Wardha 442107, India

*Corresponding author

DA: danielatmwu@gmail.com

Abstract

Introduction: Globally, the prevalence of refractive error was 12% (4), and visual impairment due to refractive error was 2.1%. In sub-Saharan Africa, the prevalence of refractive error and visual impairment due to refractive error was 12.6% and 3.4%, respectively. In Ethiopia, the prevalence of visual impairment due to refractive error varies from 2.5% in the Gurage zone to 12.3% in Hawassa city. Hence, this Meta-analysis aimed to summarize the pooled prevalence of visual impairment due to refractive error in Ethiopia.

Methods: A systematic search of the literature was conducted by the authors to identify all relevant primary studies. All articles on the prevalence of visual impairment due to refractive error in Ethiopia were identified through a literature search. The databases used to search for studies were PubMed, Science Direct, POPLINE, HENARI, Google Scholar, and grey literature was searched on Google until December 15, 2021. In this meta-analysis, the presence of publication bias was evaluated using funnel plots and Begg's tests at a significance level of less than 0.05. The sensitivity analysis was conducted to check for a single study's effect on the overall prevalence of refractive error.

Result: About 1664 studies were retrieved from initial electronic searches using international databases and google searches. A total number of 20,088 children and adolescents were included in this meta-analysis. The pooled prevalence of refractive error in Ethiopia using the random effect model was estimated to be 6 % (95% CI, 5-7) with a significant level of heterogeneity ($I2 = 94.4$ %; $p < 0.001$). The pooled prevalence of visual impairment due to refractive was analyzed by subtypes, and pooled prevalence was estimated to be 4%, 5.2%, and 1% for myopia, hyperopia, and astigmatism, respectively.

Conclusion: The pooled prevalence of visual impairment due to refractive error was high in Ethiopia. About one in twenty-five Ethiopian children and adolescents are affected by visual impairment due to refractive error.

Introduction

Refractive error occurs when the eye's optical system fails to adjust to bring parallel beams of light into proper focus on the fovea (1,2). Refractive error includes myopia, hyperopia, astigmatism, and presbyopia. It is the most common cause of reduced visual impairment (3). More than 43 percent of visual impairment is caused by uncorrected refractive error (4,5), which is the second leading cause of blindness (1).

Globally the prevalence of refractive error was 12% (4), and visual impairment due to refractive error was 2.1% (6). A review in India revealed the prevalence of visual impairment refractive error to be 10.8 %(7). In sub-Saharan Africa, the prevalence of refractive error and visual impairment due to refractive error was 12.6% and 3.4%, respectively (5). In Ethiopia, the prevalence of visual impairment due to refractive error varies from 2.5% in the Gurage zone (8) to 12.3% in Hawassa city (9).

The global initiative to eliminate avoidable blindness by 2020 (VISION 2020: the Right to Sight) has included RE as one of its five priority eye diseases, following epidemiological studies that have highlighted the escalating estimates of RE prevalence (10,11). Over 80% of visual impairments are preventable; therefore, reducing avoidable visual impairment remains an important international public health goal (12).

Visual impairment impacts economic growth, home care, and rehabilitation that account for a fraction of the annual monetary cost and loss of quality-adjusted life-years that can be averted by fifteen percent if the refractive error is corrected (13–15). In addition to the direct impact on quality-of-life visual impairment also affects the educational system by impairing reading speed, accuracy, and reading fluency (16).

Even if many studies were conducted in different parts of Ethiopia on visual impairment due to refractive error (2,8,9,17–35), their results were varying and uneven, for example, 1.9% in Jima, 11% in Gondar, and 7.2 % in Wolkite. Hence, this meta-analysis aimed to summarize the pooled prevalence of visual impairment due to refractive error in Ethiopia.

The findings from this meta-analysis will benefit health planners and concerned bodies to act on uncorrected refractive error and thereby reduce the prevalence of visual impairment due to modifiable refractive error in Ethiopia. The findings can also be used by various governmental and non-governmental organizations working in eye care to prioritize the problem of vision impairment in Ethiopia.

Methods

Study design and reporting

A review and meta-analysis were conducted to estimate the pooled prevalence of visual impairment due to refractive error in Ethiopia among children and adolescents. This review and meta-analysis were conducted according to the guideline of Preferred Reporting Items for Systematic Reviews and Meta-Analysis (additional file 1).

Eligibility criteria

Individual studies done in Ethiopia to determine the prevalence of visual impairment due to refractive error among children following criteria were included in the meta-analysis.

Population: All studies that had reported the prevalence of visual impairment due to refractive error among children.

Study designs: Observational studies reporting the prevalence of visual impairment due to refractive error among children were eligible for this systematic review and meta-analysis.

Publication status: published and unpublished articles were considered.

Year of publication: All publications reported until December 15, 2021, were considered.

Exclusion criteria: Studies that reported visual impairment but did not have a separate outcome for refractive error were excluded from the study.

The outcome of this systematic review and meta-analysis

Visual impairment due to refractive error: -Visually impaired children due to one of the following amblyopia, hyperopia, and astigmatism. For visual acuity impairment, the participants in the primary study were considered to be visually impaired when their VA was 6/12 or worse in “either eye.”

Search strategy

A systematic search of the literature was conducted by the authors to identify all relevant primary Studies. All articles on the prevalence of visual impairment due to refractive error in Ethiopia were identified through a literature search. The databases used to search for studies were PubMed, Science Direct, POPLINE, HENARI, Google Scholar, and grey literature was searched on Google until December 15, 2021. The following key search terms and Medical Subject Headings [MeSH] were used "refractive" OR "amblyopia" OR "astigmatism" OR "hyperopia" AND "Children" AND "Ethiopia [MeSH]" were used separately or in combination with the Boolean operator's terms "AND" and "OR" (additional file 2). Moreover, the reference lists of the retrieved studies were also scanned to access additional articles and screened against our eligibility criteria. Further Ethiopian university websites were used to search for unpublished articles.

Study selection

In this meta-analysis, all the searched articles were exported into the EndNote version X8 software, and subsequently, the duplicate articles were removed. Screening of retrieved articles titles, abstracts, and the full text was conducted independently by two review authors (DA & ZS) based on the eligibility criteria. Afterward, full-text articles were retrieved and appraised to approve eligibility. Finally, the screened articles were compiled together by the two investigators.

Risk of bias assessment

The qualities of the included studies were assessed, and the risks for biases were refereed using the Joanna Briggs Institute (JBI) quality assessment tool for the prevalence studies. Two reviewers (DA and ZS) assessed the quality of included studies independently, and a discrepancy between the two reviewers was resolved with discussion. The evaluation tool comprises nine parameters: (1) appropriate sampling frame, (2) correct sampling technique, (3) acceptable sample size, (4) study subject and location explanation, (5) appropriate data investigation, (6) use of valid methods for the identified conditions, (7) valid measurement for all participants, (8) using appropriate statistical analysis, and (9) adequate response rate (36). Failure to satisfy each parameter was scored as 1 if not 0. When the information provided was not satisfactory to assist

in deciding on a specific item, we agreed to grade that item as 1. The risks for biases were classified as either low (total score, 0 to 2), moderate (total score, 3 or 4), or high (total score, 5 to 9) (additional file 3).

Data extraction

The selected articles were carefully reviewed, and the required information for the meta-analysis was extracted and summarized using an extraction table in Microsoft Office Excel software. The data extraction was conducted by the two authors (DA and ZS) based on prespecified headings that are agreed on by discussion. The data extraction tool consists of the name of the author (s), year of publication, year of study, professionals conducting the visual examination, region, study design, study setting, study population, sample size, prevalence, and risk of bias.

Statistical methods and analysis

The extracted data were imported into STATA version 14 software for statistical analysis. The heterogeneity among all included studies was assessed by I^2 statistics and the Cochran Q test. In this meta-analysis, the tests indicate the presence of significant heterogeneity among included studies (I^2=98.5%, P-value < 0.001). Thus, a random-effects model was used to analyze the data. The pooled prevalence of visual impairment due to refractive error along their corresponding 95% CI was presented using a forest plot. Subgroup analyses were conducted using the region of the primary study, year of publication, year of study, study setting, sample size, eye examiners' profession, and risk of bias. Meta-regression analysis evaluated the association between the prevalence of refractive error and publication year, study year, and sample size among the included studies. In this meta-analysis, the presence of publication bias was evaluated using funnel plots and Begg's tests at a significance level of less than 0.05. The sensitivity analysis was conducted to check for a single study's effect on the overall prevalence of refractive error.

Results

Description of included studies

About 1664 studies were retrieved from initial electronic searches using international databases and google searches. The database included PubMed (n=16), HENARI (n=266), POPLINE(n=235) Science Direct (n = 46), Scopus(n=157), Google Scholar (n= 942), and Addis Ababa University repository (n = 2) studies. Of these, 1470 duplicates were removed, the

remaining 194 articles were screened by title, and 131 articles were excluded after reading their titles. Sixty-three full-text articles remained and were further assessed for their eligibility. Finally, based on the pre-defined inclusion and exclusion criteria, a total of 22 articles were included in the meta-analysis, and data were extracted for the final analysis (Fig.1).

Characteristics of the included studies

A total number of 20,088 children and adolescents were included in this meta-analysis. All included studies are cross-sectional in design. The latest article was conducted in 2020, and the earliest study was concluded in 2000. Depending on sample size, sixteen studies have a sample size greater than or equal to 500, and six studies have a sample size less than 500. Nine studies were conducted in the Amhara region, seven in the Southern Nation and Nationality Region (SNNR), three in Addis Ababa, two in Tigray, and one in the Oromia region. About fifteen were school-based, five were community-based, and two were hospital-based studies. Concerning the risk of bias among studies, the majority (16) of studies were judged to have a low risk of bias (Table 1).

The publication biases

The presence of publication bias was evaluated using funnel plots and Begg's tests at a significance level of less than 0.05. The findings revealed that publication bias was not significant for the studies on the prevalence of refractive error in Ethiopia (p =0.096) (fig.2).

Sensitivity analysis

The presence of a single study effect on the pooled prevalence of refractive error was tested using meta influence analysis and revealed that there is no study significantly affecting the pooled prevalence of refractive error in Ethiopia (Fig. 3).

Prevalence of refractive error in Ethiopia.

The pooled prevalence of refractive error in Ethiopia using the random effect model was estimated to be 6 % (95% CI, 5-7) with a significant level of heterogeneity (I^2 = 94.4 %; p < 0.001) (Fig.4).

The pooled prevalence of visual impairment due to refractive was analyzed by subtypes, and pooled prevalence was estimated to be 4% (fig. 5), 2% (fig. 6), and 1% (fig .7) for myopia,

astigmatism, and hyperopia, respectively. The heterogeneity was shown to be 94.6% for myopia, 60 % for astigmatism, and 78.8% for hyperopia.

Subgroup analysis was conducted by region, year of publication, year of study, sample size, professional diagnosing refractive error, study setting, and risk bias of studies. The sub-group conducted by region identified a lower prevalence of refractive error among studies conducted in Addis Ababa (4%), and the heterogeneity was decreased to 0% among studies conducted in Addis Ababa and the Tigray region. The pooled prevalence of refractive error was not varied by the year of publication, but the heterogeneity was decreased to 60% among studies published from 2011 to 2015. Similarly, the pooled prevalence was not varied by the year of data collection, but the heterogeneity is shown to decrease significantly (53%) among studies conducted between 2000-and 2010. Subgroup analysis conducted on the pooled prevalence of refractive error was highest among studies with a sample size between 500-750 (7%). The lowest result was identified among studies with a sample size greater than 750 (5%). In this subgroup, the heterogeneity varied from 67.0 % among studies with a sample size between 751-1000 to 97.1% among studies with a sample size between 500-750. The pooled prevalence of refractive error was found to be 4% (95% CI; 3- 6) among community-based studies. The heterogeneity decreased from 96% to 0 % after subgroup analysis based on the study setting. The pooled prevalence of refractive error was identified to be 7% among studies where refractive error diagnosis was made by an optometrist and 4% among studies where an ophthalmologist made it. In this subgroup, the heterogeneity decreased to 68% among studies where an ophthalmologist made the diagnosis, while it was 95.2 % among studies where the diagnosis was made by an optometrist (Table 2.)

Discussion

This meta-analysis is the first pooled prevalence of visual impairment due to refractive error in Ethiopia. The pooled prevalence of visual impairment due to refractive error was found to be 6 % in Ethiopia, and myopia was identified as the commonest cause of visual impairment due to refractive error. The current funding is higher than the pooled prevalence of visual impairment due to refractive error reported by a global meta-analysis conducted in 2004 (1%) (38). The difference might be explained by the variation of the study period of global metanalysis, which included studies conducted before 2004. The finding of this meta-analysis was also higher than

other global meta-analyses conducted in 2010, which has revealed the prevalence to be 2.1%(6). The difference might be from sociodemographic and eye care service differences among different countries included in the analysis.

Subgroup analysis by region in this meta-analysis showed the lowest prevalence of refractive error among studies conducted in Addis Ababa. The difference among the regions may be due to variation in universal health coverage among different regions of Ethiopia. The universal health coverage was 52.2 % in Addis Ababa, while 26% in the Oromia region (39).

Subgroup analysis conducted on the pooled prevalence of visual impairment due to refractive error was highest among studies with a sample size between 500-750. At the same time, the lowest result was identified among studies with a sample size greater than 750. This difference may be because a small sample size has an overestimation effect compared with large sample size studies (40). Further studies with a low sample size have a higher margin of error, and a large margin of error elongates the width of the confidence interval and gives an imprecise measure of the common effect size (41).

The pooled prevalence of visual impairment due to refractive error was found to be higher among studies where an optometrist made refractive error diagnosis than among studies where refractive error diagnosis was made by an ophthalmologist. This can be explained by the difference in the skill of professionals in making an appropriate diagnosis of refractive errors. Some studies reported that diagnoses of ocular disease made by optometrists are shown to vary from a diagnosis made by an ophthalmologist (42–45).

Generally, the current systematic review and meta-analysis reveal the prevalence of refractive error in Ethiopia, giving essential evidence for policymakers, clinicians, and other concerned entities who have previously overlooked the burden of refractive error on visual impairment.

Conclusion

In Ethiopia, the pooled prevalence of visual impairment due to refractive error was high. About one in twenty-five Ethiopian children and adolescents are affected by visual impairment due to myopia. Since the refractive error is a correctable cause of visual impairment, it is important to have a national figure in Ethiopia. Recognizing a high prevalence of visual impairment due to

refractive error in Ethiopia may prompt policymakers to implement effective control and prevention initiatives to reduce the refractive error burden in Ethiopia.

Declarations

The authors declare that there is no conflict of interest

Ethics approval and consent to participate

Not applicable

Consent for publication

Not applicable

Availability of Data and materials

The part of the data analyzed during this study is included in this manuscript. Other data will be available from the corresponding author upon a reasonable request.

Competing interests

The authors declare no competing interests in this work.

Funding

Nil

Authors' contributions

All authors made a significant contribution to the work reported, whether that is in the conception, study design, execution, acquisition of data, analysis, and interpretation, or in all these areas; they took part in drafting, revising, or critically reviewing the article; gave final approval of the version to be published; have agreed on the journal to which the article has been submitted; and agree to be accountable for all aspects of the work.

Reference

1. Alemayehu AM, Belete GT, Adimassu NF. Knowledge, attitude, and associated factors among primary school teachers regarding refractive error in school children in Gondar city, Northwest Ethiopia. PLoS One. 2018;13(2):31–40.

2. Sewunet SA, Aredo KK, Gedefew M. Uncorrected refractive error and associated factors among primary school children in Debre Markos District, Northwest Ethiopia. BMC Ophthalmol. 2014;14(1):1–6.

3. Zhang Q. Genetics of Refraction and Myopia [Internet]. 1st ed. Vol. 134, Progress in Molecular Biology and Translational Science. Elsevier Inc.; 2015. 269–279 p. Available from: http://dx.doi.org/10.1016/bs.pmbts.2015.05.007

4. Hashemi H, Fotouhi A, Yekta A, Pakzad R, Ostadimoghaddam H, Khabazkhoob M. Global and regional estimates of prevalence of refractive errors: Systematic review and meta-analysis. J Curr Ophthalmol [Internet]. 2018;30(1):3–22. Available from: https://doi.org/10.1016/j.joco.2017.08.009

5. Naidoo K, Kempen JH, Gichuhi S, Braithwaite T, Casson RJ, Cicinelli MV, et al. Prevalence and causes of vision loss in sub-Saharan Africa in 2015: Magnitude, temporal trends and projections. Br J Ophthalmol. 2020;104(12):1658–68.

6. Naidoo KS, Leasher J, Bourne RR, Flaxman SR, Jonas JB, Keeffe J, et al. Global vision impairment and blindness due to uncorrected refractive error, 1990Y2010. Optom Vis Sci. 2016;93(3):227–34.

7. Sheeladevi S, Seelam B, Nukella PB, Modi A, Ali R, Keay L. Prevalence of refractive errors in children in India: a systematic review. Clin Exp Optom. 2018;101(4):495–503.

8. Woldeamanuel GG, Biru MD, Geta TG, Areru BA. Visual impairment and associated factors among primary school children in gurage zone, Southern Ethiopia. Afr Health Sci. 2020;20(1):533–42.

9. Alem KD, Gebru EA. A cross-sectional analysis of refractive error prevalence and associated factors among elementary school children in Hawassa, Ethiopia. J Int Med Res. 2021;49(3).

10. Malla OK. Vision 2020: the right to sight. Kathmandu Univ Med J (KUMJ). 2004;2(1):2.

11. Naidoo KS, Jaggernath J. Uncorrected refractive errors. Indian J Ophthalmol. 2012;60(5):432–7.

12. Moyegbone JE, Nwose EU, Nwajei SD, Odoko JO, Agege EA, Igumbor EO. Epidemiology of visual impairment: focus on Delta State, Nigeria. Int J Community Med Public Heal. 2020;7(10):4171.

13. Marques AP, Ramke J, Cairns J, Butt T, Zhang JH, Muirhead D, et al. Global economic productivity losses from vision impairment and blindness. EClinicalMedicine [Internet]. 2021;35:100852. Available from: https://doi.org/10.1016/j.eclinm.2021.100852

14. Holden BA, Fricke TR, Wilson DA, Jong M, Naidoo KS, Sankaridurg P, et al. Global Prevalence of Myopia and High Myopia and Temporal Trends from 2000 through 2050. Ophthalmology [Internet]. 2016;123(5):1036–42. Available from: http://dx.doi.org/10.1016/j.ophtha.2016.01.006

15. Stein JD. Economic impact of visual impairment and blindness in the United States. Evidence-Based Ophthalmol. 2007;8(4):240–1.

16. Ramani KK, Police S, Jacob N. Impact of low vision care on reading performance in children with multiple disabilities and visual impairment. Indian J Ophthalmol. 2014;62(2):111–5.

17. Hailu Y, Hiko D, Shaweno T. Prevalence of visual impairment and associated factors among primary schoolchildren in Addis Ababa, Central Ethiopia. Clin Ophthalmol. 2020;14:767–74.

18. Mehari ZA, Yimer AW. Prevalence of refractive errors among schoolchildren in rural central Ethiopia. Clin Exp Optom. 2013;96(1):65–9.

19. Merrie YA, Tegegne MM, Munaw MB, Alemu HW. Prevalence and associated factors of visual impairment among school-age children in Bahir Dar City, Northwest Ethiopia. Clin Optom. 2019;11:135–43.

20. Kedir J, Girma A. Prevalence of refractive error and visual impairment among rural school-age children of Goro District, Gurage Zone, Ethiopia. Ethiop J Health Sci. 2014;24(4):353–8.

21. Dhanesha U, Polack S, Bastawrous A, Banks LM. Prevalence and causes of visual impairment among schoolchildren in Mekelle, Ethiopia. Cogent Med [Internet].

2018;5(1):1554832. Available from: https://doi.org/10.1080/2331205X.2018.1554832

22. Asferaw M, Woodruff G, Gilbert C. Causes of severe visual impairment and blindness in students in schools for the blind in Northwest Ethiopia. BMJ Glob Heal. 2017;2(2):1–8.

23. Demissie BS, Solomon AW. Magnitude and causes of childhood blindness and severe visual impairment in Sekoru District, Southwest Ethiopia: A survey using the key informant method. Trans R Soc Trop Med Hyg [Internet]. 2011;105(9):507–11. Available from: http://dx.doi.org/10.1016/j.trstmh.2011.04.007

24. Demissie BS DE. Original Article Patterns of Eye Diseases in Children Visiting a Tertiary Teaching Hospital : Ethiopia. Ethiop J Heal Sci. 2014;24(1):69–73.

25. Belete GT, Anbesse DH, Tsegaye AT, Hussen MS. Prevalence and associated factors of myopia among high school students in Gondar town, northwest Ethiopia, 2016. Clin Optom. 2017;9:11–8.

26. Gessesse SA, Teshome AW. Prevalence of myopia among secondary school students in Welkite town: South-Western Ethiopia. BMC Ophthalmol. 2020;20(1):2–7.

27. Shaffi M, Bejiga A. Common eye diseases in children of rural community in Goro district, Central Ethiopia. Ethiop J Heal Dev. 2005;19(2).

28. Mehari ZA. Pattern of childhood ocular morbidity in rural eye hospital, Central Ethiopia. BMC Ophthalmol. 2014;14(1).

29. Zelalem M, Abebe Y, Adamu Y, Getinet T. Prevalence of visual impairment among school children in three primary schools of Sekela Woreda, Amhara regional state, north-west Ethiopia. SAGE Open Med. 2019;7:205031211984976.

30. Tibebu Kassa GDA. prevalance of refractive error in preschool and school childern of Debark and Kola Diba towns,Northwest Ethiopia. Ethiopian Journal of Health Development. 2000. p. 117–24.

31. Darge HF, Shibru G, Mulugeta A, Dagnachew YM. The Prevalence of Visual Acuity Impairment among School Children at Arada Subcity Primary Schools in Addis Ababa, Ethiopia. J Ophthalmol. 2017;2017:10–7.

32. Ferede AT, Alemu DS, Gudeta AD, Alemu HW, Melese MA. Visual Impairment among Primary School Children in Gondar Town, Northwest Ethiopia. J Ophthalmol. 2020;2020.

33. Worku M, Gizaw Z, Kassahun Belew A, Wagnew A, Hunegnaw MT. Prevalence and Associated Factors of Overweight and Obesity among High School Adolescents in Bahir Dar City, Northwest, Ethiopia: A Cross-Sectional Study. J Obes. 2021;2021:143–53.

34. Yared AW, Belaynew WT, Destaye S, Ayanaw T, Zelalem E. Prevalence of refractive errors among school children in Gondar town, northwest Ethiopia. Middle East Afr J Ophthalmol. 2012;19(4):372–6.

35. Bezabih L, Abebe TW, Fite RO. Prevalence and factors associated with childhood visual impairment in Ethiopia. Clin Ophthalmol. 2017;11:1941–8.

36. Joanna Briggs Institute. Checklist for Prevalence Studies. Checkl prevalance Stud [Internet]. 2016;7. Available from: http://joannabriggs.org/assets/docs/critical-appraisal-tools/JBI_Critical_Appraisal-Checklist_for_Prevalence_Studies.pdf.

37. Assem AS, Tegegne MM, Fekadu SA. Prevalence and associated factors of myopia among school children in Bahir Dar city Northwest Ethiopia, 2019. PLoS One [Internet]. 2021;16(3 March):14–8. Available from: http://dx.doi.org/10.1371/journal.pone.0248936

38. Resnikoff S, Pascolini D, Mariotti SP, Pokharel GP. Global magnitude of visual impairment caused by uncorrected refractive errors in 2004. Bull World Health Organ. 2008;86(1):63–70.

39. Eregata GT, Hailu A, Memirie ST, Norheim OF. Measuring progress towards universal health coverage: National and subnational analysis in Ethiopia. BMJ Glob Heal. 2019;4(6):1–9.

40. Faber J, Fonseca LM. How sample size influences research outcomes. Dental Press J Orthod. 2014;19(4):27–9.

41. Liu XS. Sample Size and the Precision of the Confidence Interval in Meta-analyses. Ther Innov Regul Sci. 2015;49(4):593–8.

42. Hau SCH, Ehrlich D, Binstead K, Verma S. An evaluation of optometrists' ability to

correctly identify and manage patients with ocular disease in the accident and emergency department of an eye hospital. Br J Ophthalmol. 2007;91(4):437–40.

43. COCKBURN DM, GUTTERIDGE IF. Reasons for Referrals by Optometrists to Ophthalmologists: An Audit of Ocular Disease Detection. Aust J Optom. 1980;63(1):13–8.

44. Cockburn DM, Gufteridge IF, Dougkrs IS. An audit of disease diagnoses made by optometrists. Clin Exp Optom. 1994;77(2):47–57.

45. Tong L, Saw SM, Tan D, Chia KS, Chan WY, Fiacle, et al. Sensitivity and specificity of visual acuity screening for refractive errors in school children. Optom Vis Sci. 2002;79(10):650–7.

Table 1: characteristics of included studies for pooled prevalence of visual impairment due to refractive error in Ethiopia.

Author name	Year of publication	Region	Study design	Sample size	Risk of bias	SE	Prevalence of RE	Study setting	Diagnosed by	year of study
Zelalem et al (29).	2019	Amhara	Cs	875	low	0.0065	0.038	Community based	Optometrist	2016
Sewunet et al(21).	2014	Amhara	Cs	420	low	0.0138	0.088	School based	Optometrist	2013
Kassa and Alene (30)	2004	Amhara	Cs	1134	low	0.0078	0.076	School based	Not mentioned	2000
Weldeamanuel et al(8).	2020	SNNP	Cs	1064	low	0.0048	0.025	School based	Ophthalmologist	2017
Alem and Gebru(9)	2021	SNNP	Cs	554	low	0.0047	0.123	School based	Optometrist	2016
Darge et al(31).	2017	Addis Ababa	Cs	378	medium	0.011	0.05	School based	Ophthalmologist	2015
Ferede et al(32).	2020	Amhara	Cs	1289	medium	0.0044	0.026	School based	Ophthalmologist	2016
Assem et al(33).	2021	Amhara	Cs	601	medium	0.0113	0.085	School based	Optometrist	2019
Yared et al(34).	2012	Amhara	Cs	1852	low	0.005	0.049	School based	Optometrist	2010
Bezabih et al (35).	2017	Addis Ababa	Cs	718	medium	0.0069	0.036	School based	Optometrist	2016
Hailu et al(17).	2020	Addis Ababa	Cs	773	medium	0.0071	0.041	School based	Ophthalmologist	2019
Mehari and Yimer(18).	2012	SNNP	Cs	4238	medium	0.0037	0.063	School based	Optometrist	2010
Kedir and Girma(20).	2010	SNNP	Cs	570	low	0.0077	0.035	Community based	Not mentioned	2018
Dhanesha et al(21).	2018	Tigray	Cs	1137	low	0.0069	0.058	School based	Not mentioned	2012
Mehari et al (28).	2014	SNNP	Cs	735	low	0.0083	0.053	Hospital based	Ophthalmologist	2003
Shaffi and Bejiga(27).	2005	SNNP	Cs	826	low	0.0084	0.063	Community based	Not mentioned	2003
Demissie and Demissie(24).	2014	Oromia	Cs	341	low	0.0128	0.059	Hospital based	Optometrist	2018

Belete et al(25).	2016	Amhara	Cs	495	low	0.0141	0.11	School based	Optometrist	2016
Gessese and Teshome(26).	2020	SNNP	Cs	1271	low	0.0073	0.072	School based	Optometrist	2016
Demissie et al(23).	2011	Oromia	Cs	112	low	0.0212	0.053	School based	Ophthalmologist	2010
Asferaw et al(22).	2017	Oromia	Cs	104	low	0.0134	0.019	Community based	Not mentioned	2015
Tegegne et al(19).	2021	Amhara	Cs	601	low	0.0101	0.065	Community based	Optometrist	2017

table 2 Click here to access/download;Figure;table 2.docx

Table 2: Sub-group analysis by region, year of publication, year of study, professional diagnosed, study setting and sample size.

**Sub-group analysis**	**VI due to RE%**	**95% confidence interval**	**Heterogeneity (I^2%)**	**P-value**
**Sub-group analysis by year of publication**				
1. < 2011	6.0	4.0 – 8.0	79.5	P = 0.002
2. 2011-2016	6.0	5.0 – 7.0	60.6	P = 0.038
3. 2017-2021	6.0	4.0 – 7.0	96.5	P < 0.001
**Sub-group analysis by year of study**				
1. 2000-2010	6.0	4.0 – 8.0	95.8	P< 0.001
2. 2011-2021	6.0	5.0 – 7.0	53.0	P = 0.059
**Sub-group analysis by professional who diagnosed RE**				
1. Ophthalmologist	4.0	3.0 – 5.0	68.0	P= 0.008
2. Optometrist	7.0	2.0 – 9.0	95.2	P < 0.001
**Sub-group analysis by regions of Ethiopia**				
1. Amhara region	6.0	5.0 – 6.0	91.4	P < 0.001
2. Oromia	4.0	2.0 – 7.0	59.8	P < 0.083
3. Addis Ababa	4.0	3.0 – 5.0	0.0	P = 0.556
4. Tigray	6.0	4.0 – 7.0	-	-
8. Southern Nation Nationality Region of Ethiopia	6.0	3.0 – 9.0	97.5	P < 0.001
**Sub-group analysis by study setting**				
1. Hospital based	5.0	4.0 – 7.0	0.0	P < 0.069
2. School based	6.0	5.0 – 8.0	96.2	P < 0.001
3. Community based	4.0	3.0 – 6.0	73.5	P < 0.004
**Sub-group analysis by sample size**				
1. <500	6.0	4.0 – 9.0	81.4	P < 0.001
2. 501-750	7.0	3.0 – 10.0	97.1	P < 0.001
3. 751-1000	5.0	3.0 – 6.0	67.0	P=0.048
4. 1001-1250	5.0	2.0 – 8.0	94.5	P < 0.001
5. >1250	6.0	5.0 –7.0	94.4	P < 0.001

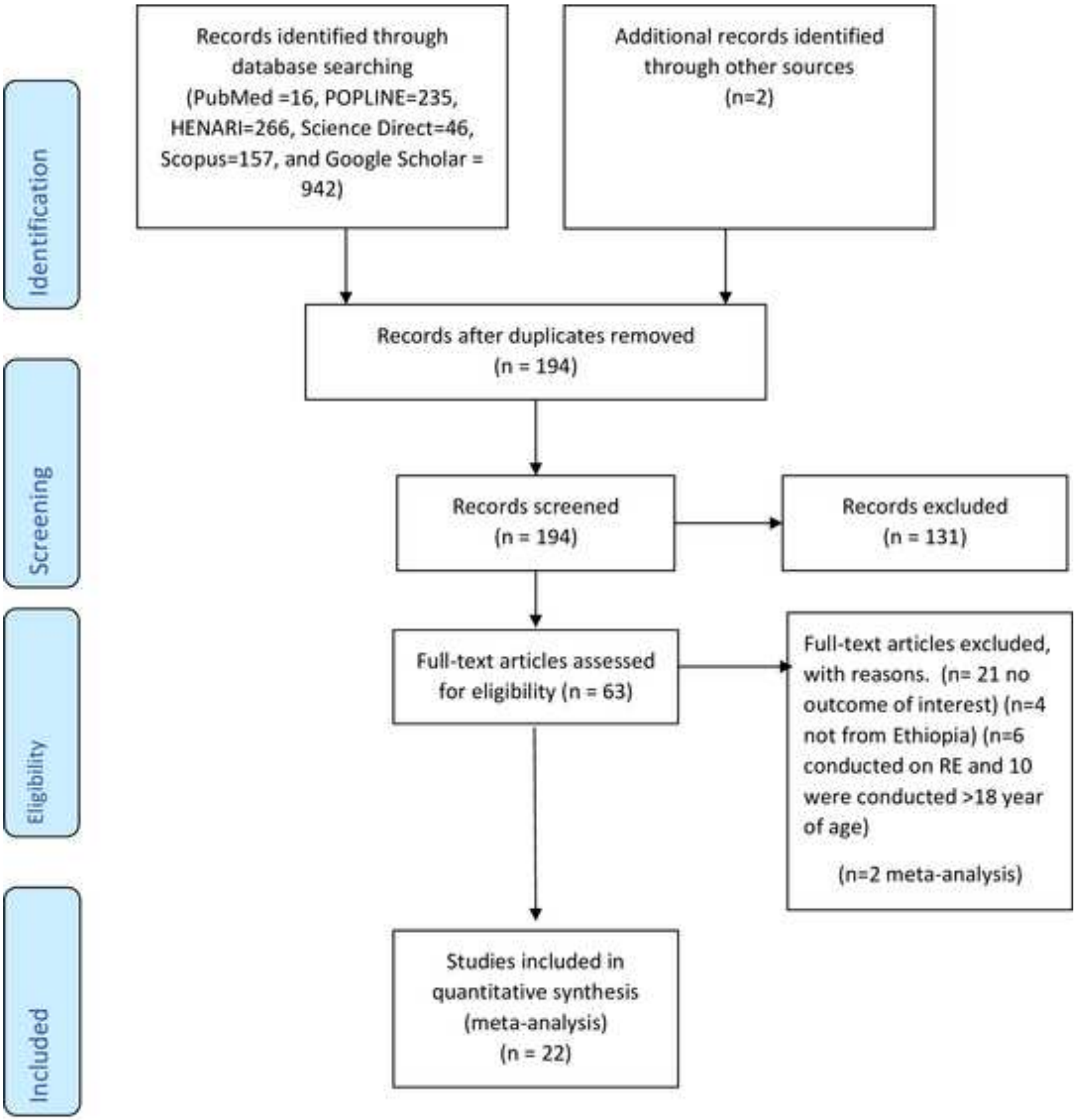

Figure 1: Flow diagram of systemic review and meta-analysis conducted on visual impairment due to refractive error in Ethiopia, 2021.

Figure 2,3

Click here to access/download;Figure;figure .2.tif

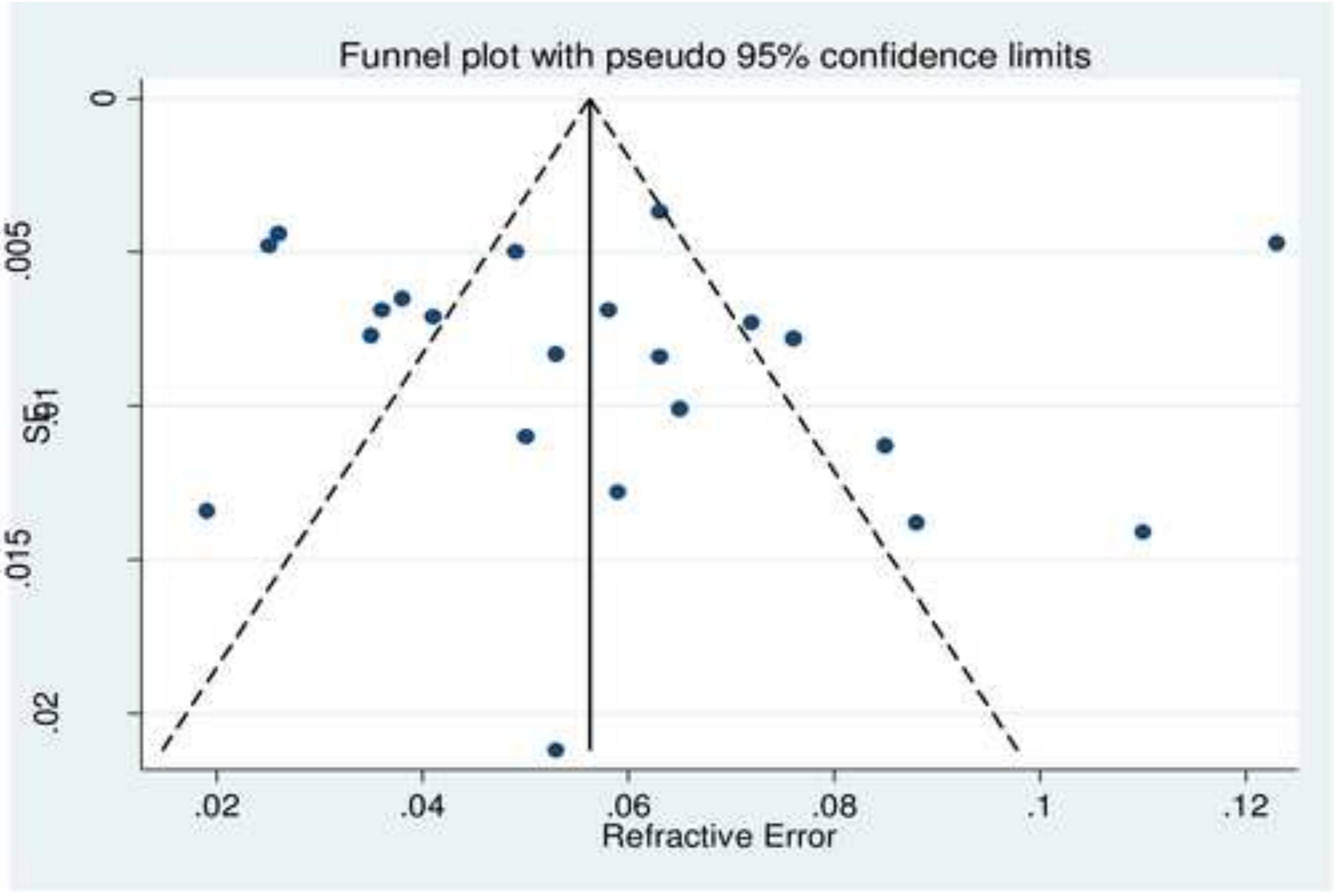

Figure 2: Showing the publication bias of studies included for meta-analysis of visual impairment due to refractive error in Ethiopia.

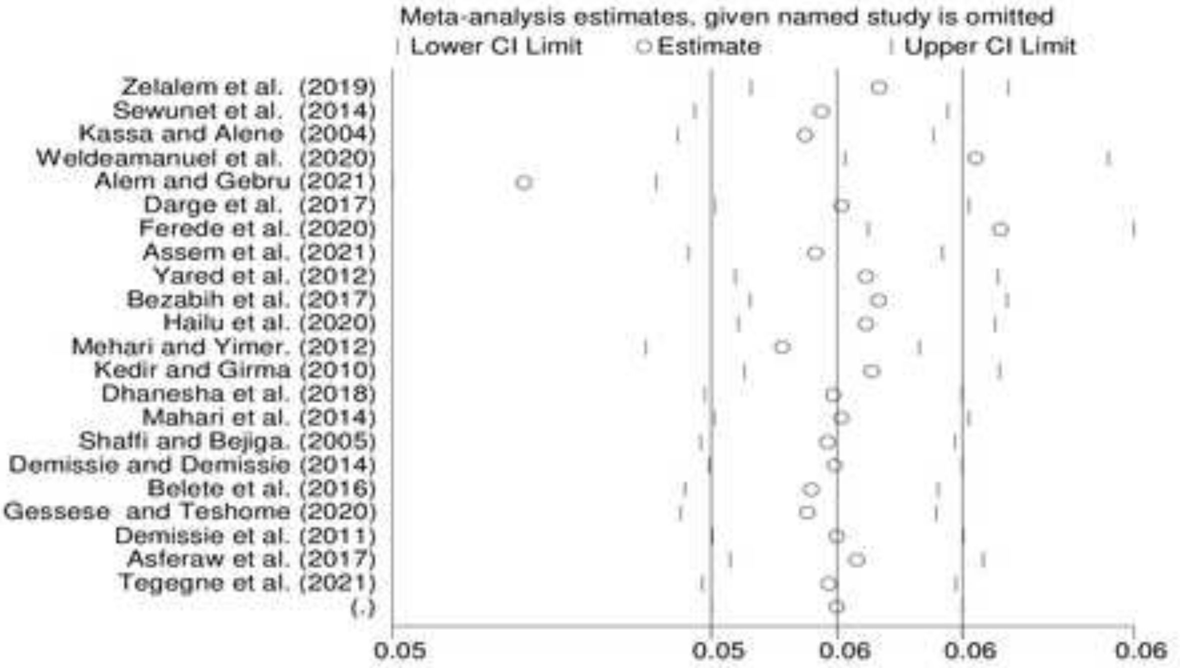

Figure 3: Showing the sensitivity analysis of meta-analysis of visual impairment due to refractive error in Ethiopia.

Figure 4

Click here to access/download;Figure;figure 4.tif

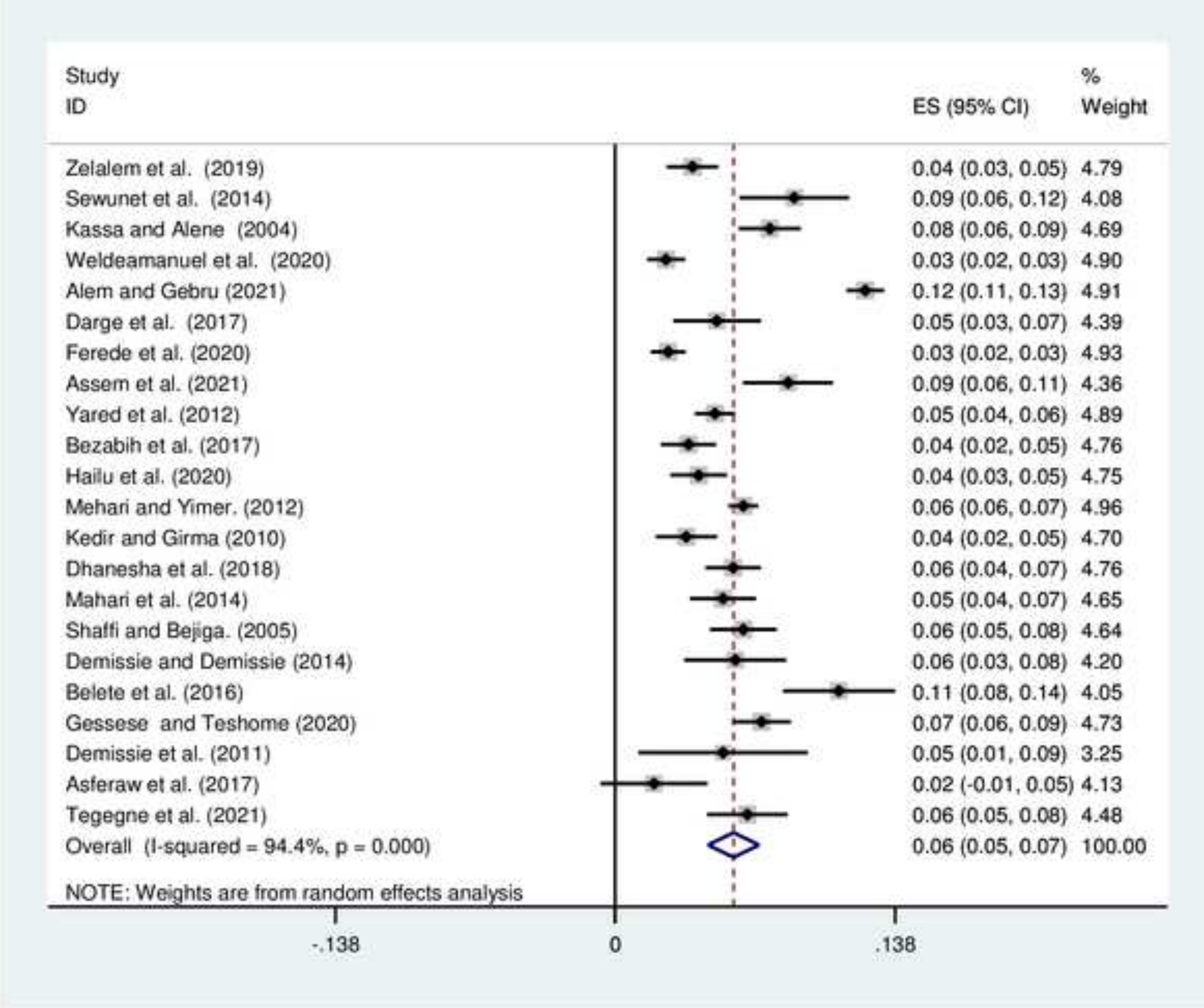

Figure 4: Funnel plot showing pooled prevalence of visual impairment due to refractive error in Ethiopia.

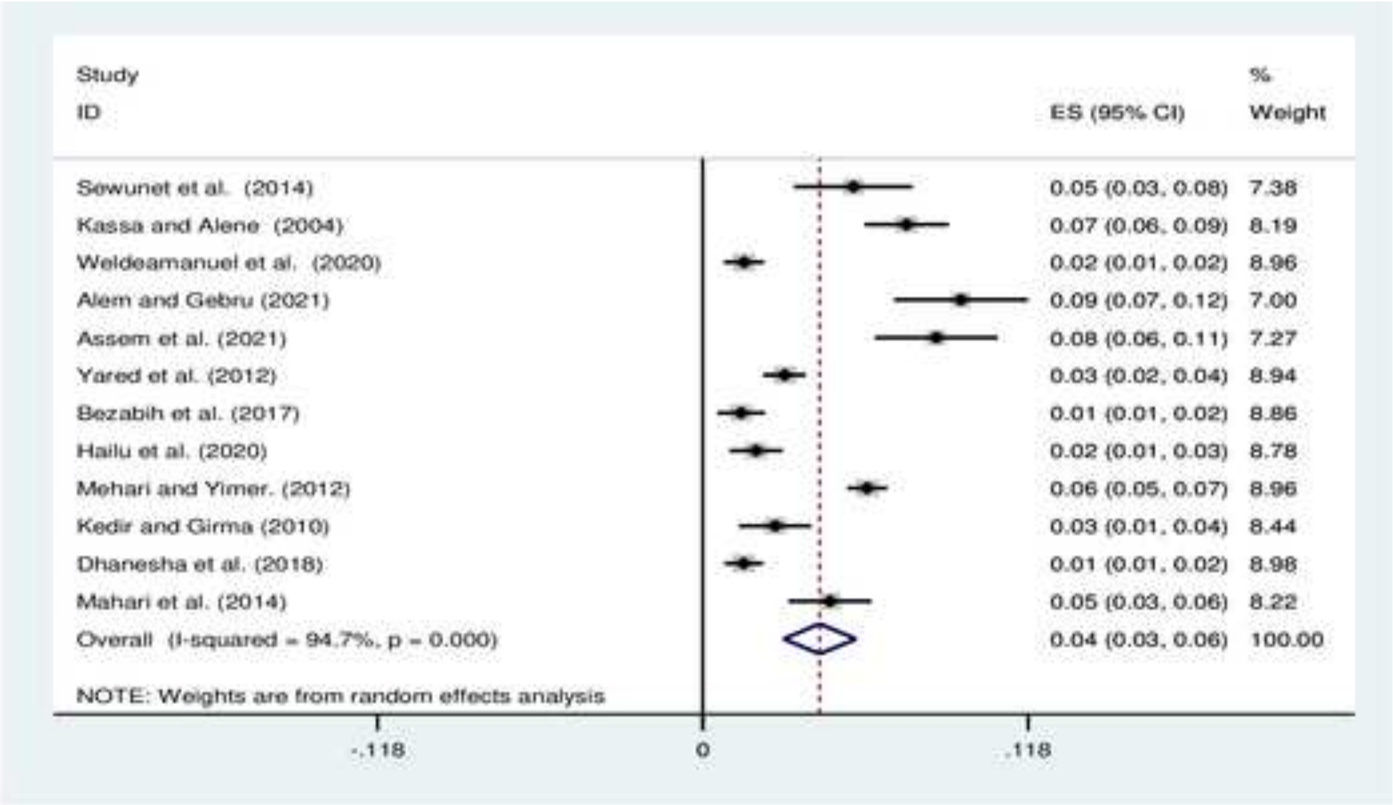

Figure 5: pooled prevalence of visual impairment due to myopia in Ethiopia.

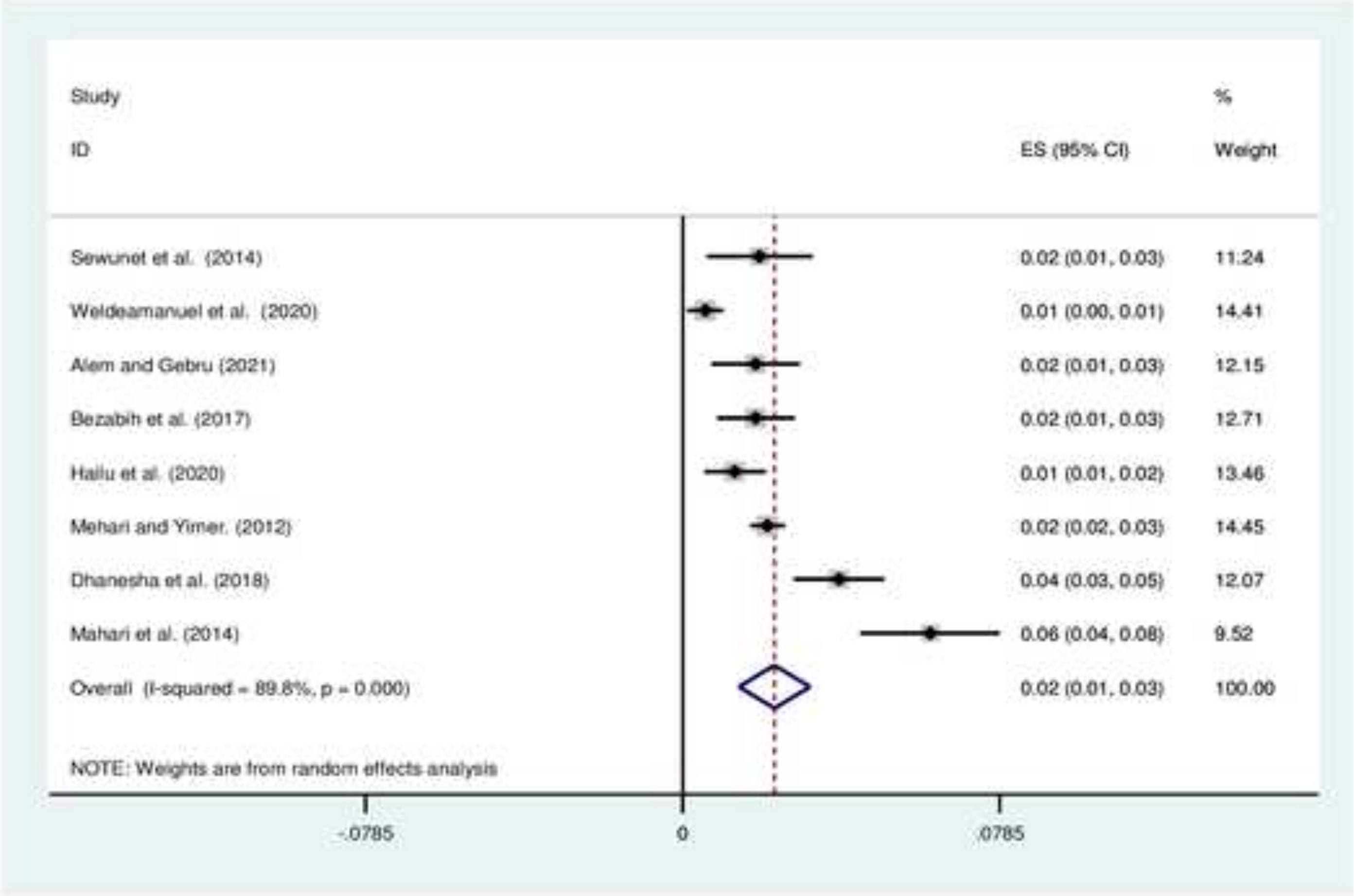

Figure 6: pooled prevalence of visual impairment due to astigmatism in Ethiopia

Figure 7

Click here to access/download;Figure;Figure .7.tif

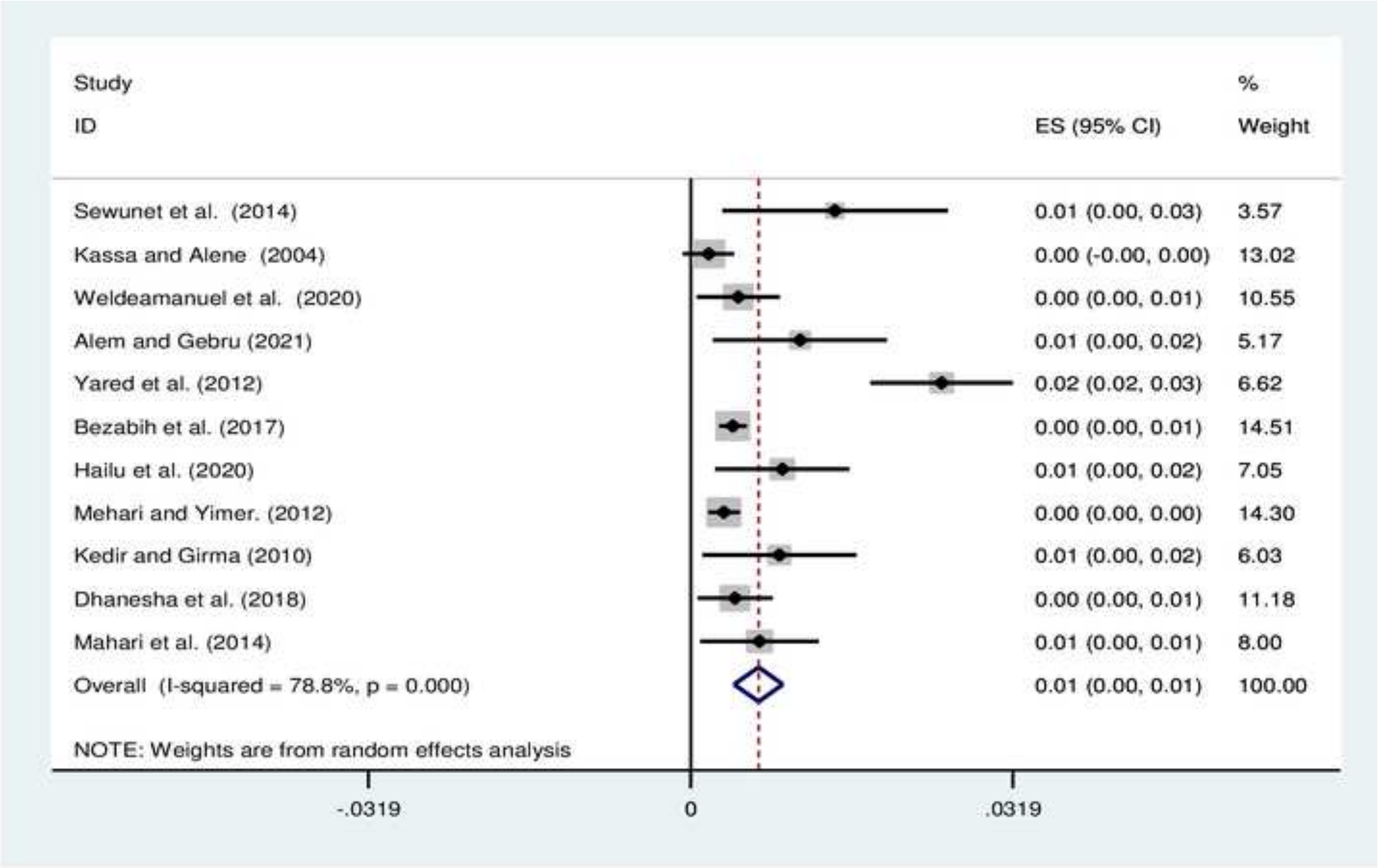

Figure 7 : pooled prevalence of visual imparment due to hyperopia in Ethiopia.

Click here to access/download
Supporting Information
PRISMA_2020_checklist.docx

Click here to access/download
Supporting Information
additional file 2.docx

Click here to access/download
Supporting Information
additional file 3.docx
